# Acute Effect of a Protein Supplement on Targeted Plasma Amino Acid Profile among Healthy Asian Indians: A Randomized Controlled Trial

**DOI:** 10.1155/2020/8946820

**Published:** 2020-05-27

**Authors:** Ammu Kurien, Nidhi Sharma, Madhavi Marathe, Nandan Joshi, Sumithra Selvam, Sarita Devi, Sucharita Sambashivaiah

**Affiliations:** ^1^Division of Nutrition, St. John's Research Institute, Bengaluru, India; ^2^Health Care Nutrition Science & Medical Affairs, Nutricia International Pvt. Ltd., Mumbai, India; ^3^Division of Epidemiology, Biostatistics and Population Health, St. John's Research Institute, Bengaluru, India; ^4^Department of Physiology, St. John's Medical College, Bengaluru, India

## Abstract

**Background:**

Indians have a poor protein intake in terms of quantity as well as quality because of their predominantly cereal-based diet. However, there is limited information on circulatory amino acid levels in healthy Indians. Herein, we evaluated the acute effect of a protein supplement on the plasma levels of essential amino acids (EAAs) in healthy Indian adults, using targeted EAA analysis.

**Methods:**

In this double-blind, randomized, crossover study, 20 healthy Indian adults were randomized to receive the test protein supplement (treatment arm, *n* = 10) or placebo (control arm, *n* = 10) with milk, after overnight fasting. After 7 days, the participants returned for the crossover treatment. Blood samples were collected at baseline and at 60 and 120 min after protein/placebo consumption. Plasma EAA levels were estimated using liquid chromatography-tandem mass spectrometry. Repeated measures ANOVA was performed to assess the effect of treatment on EAA levels. *P* values < 0.05 were considered statistically significant.

**Results:**

At baseline, mean plasma levels did not differ significantly between the two arms for any of the EAAs. In the treatment arm, the mean levels of all EAAs increased significantly from baseline to 60 min (*P* < 0.01), with no significant change from 60 to 120 min. There was no significant change in amino acid levels in the control arm. The magnitude as well as percentage of increase from baseline to 60 min was significantly greater in the treatment arm than in the control arm for all EAAs.

**Conclusion:**

Compared to placebo, protein supplement increased circulatory amino acid levels in healthy Indians. The observed increase in EAA levels and its role in conjunction with exercise in both healthy and diseased states need to be further evaluated. This is the first dataset exploring targeted EAA profiles and the effect of a protein supplement among healthy Indians. The clinical trial is registered with CTRI/2018/12/016777.

## 1. Introduction

Indians follow a predominantly vegetarian diet that does not meet the recommended daily allowance of protein [1–3]. It has been estimated that 60% of the protein Indians consume in their diet comes from cereals, which have low digestibility and are an incomplete protein source. Cereals do not provide all essential amino acids as they are deficient in lysine [3, 4]. Therefore, it is a matter of concern that Indians consume a diet that is poor not only in terms of quantity but also in terms of quality.

Dietary proteins should provide the nine essential amino acids (EAAs) in adequate quantities to allow for the synthesis of tissue proteins. The appropriate proportions and quantities of EAAs in food determine the quality of the protein source [3]. Three important parameters affect the quality of a protein: the protein characteristics, the food matrix in which it is consumed, and the characteristics of the consumer (e.g., age, health status, physiological status, and energy balance) [5].

Equally, it is important to understand dietary protein quality [6]. Good quality protein provides EAAs, especially branched-chain amino acids (BCAAs: leucine, isoleucine, and valine), which are needed for the synthesis of muscle protein. Thus, the protein quality is important to gain and maintain muscle mass [7]. Preservation of skeletal muscle mass is of great importance for maintaining metabolic health, functional capacity, and quality of life [8].

Protein stimulates muscle protein synthesis at rest and during exercise recovery [9]. In the fed state, protein synthesis in the skeletal muscle accounts for more than 50% of the total protein synthesis occurring in the whole body [10]. During the recovery phase after resistance exercise, an increase in the rates of amino acid transport into muscle cells may contribute to muscle anabolism by increasing amino acid availability for protein synthesis [11]. It is important to understand the role of the amino acid pool in protein homeostasis.

As an alternative to a meat-based diet, dairy products are the most commonly consumed protein source among Asian Indians [12]. In a recently published systematic review on the impact of milk intake on exercise performance and recovery of muscle function, mixed results have been obtained. While some studies found a significant positive effect of milk on exercise performance and recovery of muscle function, others did not find any effect. This might be attributed to the heterogeneity among the included studies with respect to milk ingestion (amount consumed, the timing of consumption, etc.), type of intervention, and the outcomes measured [13]. Nevertheless, milk is a source of protein and may lead to an increase in the serum amino acid concentration, thereby facilitating the repair of muscle damage. However, milk alone might not be enough to meet the daily protein requirement of an individual. Therefore, an additional protein supplement combined with milk could be a convenient method of protein delivery among Asian Indians [14].

The majority of studies on protein supplements so far have focused on children [15], pregnant women [16], elderly individuals [17], patients with specific diseases [18], or athletes [19]. Luiking et al. demonstrated increased plasma levels of total amino acids, EAAs, and leucine as a result of protein supplementation in elderly individuals [17]. Consumption of protein supplements before or after exercise was also found to increase muscle protein synthesis and functionality in athletes [19]. Other benefits of protein supplementation include improvement in fetal growth among pregnant women [16], better physical growth in children [15], and a reduction in cardiovascular risk markers in patients with type 2 diabetes [18]. However, very little is known about protein supplementation and availability of amino acids among healthy, young, and middle-aged Asian Indian individuals, who form the major component of the population in India. Optimum physical performance and muscle functionality are of utmost importance for this population. Thus, the present study aimed to evaluate the acute effect of a protein supplement with milk on the plasma amino acid pool through targeted EAA analysis among healthy Indian adults.

## 2. Methods

### 2.1. Subject Recruitment

This double-blind, randomized crossover study included healthy male and female volunteers aged 20–45 years and with a body mass index (BMI) 18.5–29.9 kg/m^2^. All participants were recruited from in and around St. John's Medical College and Hospital, Bengaluru, and were screened for inclusion and exclusion criteria. The exclusion criteria included a positive history of type 2 diabetes, prediabetes, hypertension, tuberculosis, ischemic heart disease, anemia, underweight, acute weight loss, cancer, lactose intolerance, protein allergy or any food allergy, and consumption of any medication or protein supplement or a medical condition that could affect the outcome measures. The participants who were finally selected after applying the inclusion and exclusion criteria underwent tests for fasting blood sugar, glycated hemoglobin (HbA1c), total cholesterol, low-density lipoprotein, high-density lipoprotein, and triglycerides. The study protocol was approved by the institutional ethics committee (study reference no. 219/2018; CTRI registration no. CTRI/2018/12/016777) and written informed consent was obtained from all participants.

**Figure 1:**
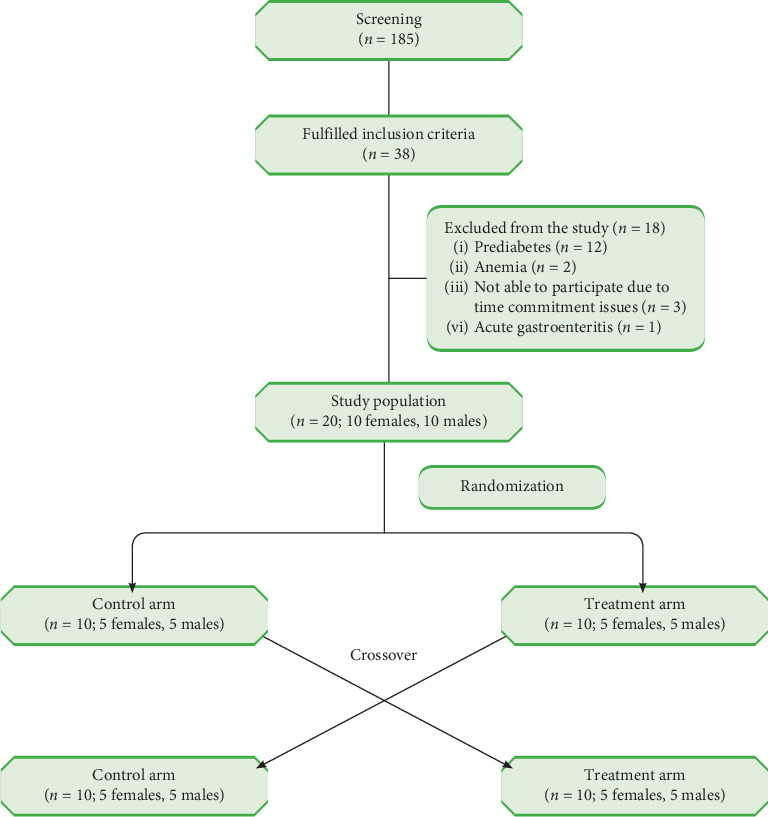
CONSORT diagram of the participant selection process.

Of the 185 individuals screened, 38 healthy volunteers fulfilled the study criteria. Following the blood investigation and clinical evaluation, participants were recruited to meet required sample size of 20. Sample size was calculated based on a study by Burke et al. with 90% power and 5% level of significance [20].  Figure 1 shows the CONSORT flowchart describing the participant enrollment. A total of 18 participants were excluded due to one of the following underlying conditions: prediabetes (*n* = 12), anemia (*n* = 2), acute gastroenteritis (*n* = 1), and time commitment issues (*n* = 3). Finally, 20 volunteers (10 males, 10 females) were randomized to either the control or treatment arm. Following a washout period of 7 days, all participants were crossed over to the other study arm. There were no dropouts following randomization and all participants completed both stages of the study.

### 2.2. Questionnaires and Body Composition

Each participant underwent a detailed clinical and anthropometric evaluation. Anthropometric measurements included body weight, height, skinfold thickness at four sites (biceps, triceps, subscapular, and suprailiac regions), and circumferences (mid-arm, waist, hip) using standard procedures. The following parameters were derived: BMI, waist-hip ratio, percentage of fat, and fat-free mass (kg) [21]. Dietary assessment was performed using 24 h food recall and a food frequency questionnaire [22]. Information about the physical activity level of the participants was collected using a physical activity questionnaire [23]. Body fat and lean mass were measured using dual-energy X-ray absorptiometry (DXA, Lunar Prodigy Advanced PA+301969 whole body scanner with software version 12.30, GE Medical Systems, USA). DXA scans were performed with the subject wearing light clothing and no metal objects. The mass of lean soft tissue, fat, and bone mineral density for the whole body and for specific regions were obtained [24].

### 2.3. Test Protein Supplement and Placebo

A test protein supplement (Protinex, Nutricia Pvt. Ltd, India) and placebo (maltodextrin-based powder, Nutricia Pvt. Ltd, India), matched for weight, color, and taste, were used for the experiment. Both weighed 35 g and were stored in similar packets. Both protein and placebo supplement were prepared by adding the product to 200 mL of lukewarm milk and then stirring until it dissolved. The containers were weighed before and after consumption and all participants were able to completely consume the beverages within 10 min of preparation. When dissolved in milk, the test protein supplement provided 244 kcal and 18 g of protein (12 g from supplement and 6 g from milk) while the placebo provided 252 kcal and 6 g of protein (from milk). The details of the test protein supplement and placebo along with amino acid profile are provided in supplementary material.

### 2.4. Experimental Protocol

The participants were randomized either to sequence AB (placebo followed by treatment) or BA (treatment followed by placebo). An online random number generator was used to allocate study arms for Visit 1 of the study. In the crossover Visit 2, the alternate arm was allocated. The study product code was provided in the randomization list. There were no restrictions while generating the random allocation sequence for the 20 subjects.

A person not involved in the study procedures possessed the randomization list and personally randomized each subject using the randomization list. The study personnel did not have access to the list. The products were packed in identical sachets which were coded. All study personnel, participants, and statistician were blinded.

On the morning of the experiment, participants reported to the laboratory in a fasting state (∼8–12 h). Body weight and blood pressure were recorded and an intravenous cannula was inserted for blood sampling. The baseline blood samples were collected in the fasting state for targeted EAA analysis. The samples were immediately kept in an icebox and centrifuged at 3,000 rpm for 10 min at 4°C (REMI cooling centrifuge, model no, C-23 BL. ISO-9001-2008, Mumbai, India). The separated plasma was stored in Eppendorf tubes at −80°C. Each participant then consumed the assigned product based on the allocated randomization code assigning him/her to either the treatment or the control arm. Those allocated to the treatment arm consumed protein supplement (35 g) with 200 mL lukewarm milk, while those allocated to the control arm were given an equal amount of placebo with milk. Blood samples were collected at 60 and 120 min after the consumption of the protein supplement or placebo for targeted EAA analysis. The samples were processed and stored at −80°C until analysis. The participants returned to the laboratory after 1 week (minimum of 7 days washout period) for the crossover treatment.

### 2.5. Targeted EAA Analysis

The levels of the nine EAAs in the collected blood samples were analyzed. Targeted EAA analysis was performed using eight levels (range: 5–1000 *µ*mol/L) of calibrators (mixed standards of isoleucine, leucine, valine, methionine, lysine, threonine, tryptophan, phenylalanine, and histidine; *>*97% purity; Sigma-Aldrich, MO, USA). A U–^2^H-labeled amino acid mix (678 *µ*mol/L; >97% purity; Cambridge Isotope Laboratories, Andover, MA) and norvaline (250 *µ*mol/L; Sigma-Aldrich, MO, USA) were used as internal standards. In-house quality control (QC) samples were prepared from pooled plasma samples obtained from multiple volunteers.

For the extraction of amino acids, the stored blood samples were thawed on ice and a 100-*µ*L aliquot of each sample was spiked with 25 *µ*L of each internal standard. Each sample was then acidified with 25 *µ*L of 2 M HCl (Merck, India) and deproteinized with 400 *µ*L of chilled acetonitrile (Merck, India), followed by vortexing and centrifugation at 12,000 rpm for 10 min at 4°C. The supernatant was transferred to preconditioned cation exchange columns (50WX8–100 ion-exchange resin, Sigma-Aldrich, MO, USA) and free amino acids were eluted with 2 mL of 4 M ammonium hydroxide (NH_4_OH, Merck, India) solution.

The eluate was dried and derivatized to its corresponding *N*-ethoxycarbonyl ethyl ester derivative [25]. The derivatized sample was then diluted with methanol (1 : 3 ratio) and 3 *µ*L was injected in a liquid chromatography-tandem mass spectrometer (LC-MS/MS, 6460 Triple Quadrupole, Agilent Technologies, CA, USA) equipped with Agilent Jet Stream technology, 1290 Infinity binary pump, and a temperature-controlled column compartment and autosampler. Amino acids were separated on a reverse-phase high-performance liquid chromatography (HPLC) phenyl-hexyl column (ZORBAX Eclipse Plus Phenyl-Hexyl column, 2.1 × 150 mm ID, 1.8 *μ*m particle size, Agilent Technologies) maintained at 45°C, with a flow rate of 0.4 mL/min. A gradient elution program was used with aqueous mobile phase A containing 0.1% formic acid in water and mobile phase B containing methanol and isopropyl alcohol (80 : 20) with 0.1% formic acid and 10 mM ammonium formate. The elution program was started with 5% B (95% A), which was increased to 25% (75% A) over 3 min. By 25 min, the proportion of B had increased to 60% (40% A) and was finally set back to the initial conditions with a hold time of 5 min. MS was performed with an electrospray ionization source in the positive polarity mode, using a dynamic multiple reaction monitoring- (DMRM-) based methods.

In-house QC samples were analyzed at the beginning and end of each analytical batch of samples. The data were processed using Agilent MassHunter Quantitative Analysis Software (version B 07.00). The intra- and interassay coefficients of variation were <10% and <15%, respectively.

### 2.6. Statistical Analysis

Descriptive statistics were reported as mean and standard deviation (SD) for normally distributed continuous variables and median and interquartile range for the rest. Categorical variables were reported as numbers and percentages. To compare the characteristics between two groups (e.g., male vs. female, treatment vs. control arms), independent *t*-tests were used for normally distributed variables, and the Mann-Whitney *U* test for nonnormally distributed variables. Repeated measures ANOVA was performed to assess the effect of the treatment on EAA parameters. The main test of interest was the within-person treatment effect, that is, time effect and interaction effect (time × treatment) adjusted for the sequence effect. The sequence effect refers to the possible effect of the order in which the participant received test protein and placebo. Participants were randomized to receive either the test protein supplement followed by placebo or *vice versa* based on the randomization codes assigned by a biostatistician. Bonferroni test was used for multiple comparisons. A *P* value < 0.05 was considered statistically significant. All the analyses were performed using SPSS version 24.0.

## 3. Results

### 3.1. Baseline Characteristics of the Study Population

All the 20 subjects recruited for the study completed the experiment. The baseline characteristics of the study participants are provided in Table 1. The mean age and BMI of the study subjects were 28.6 ± 6.4 years and 22.9 ± 2.63, respectively. The minimum, maximum, and mean (±SD) plasma levels of all EAAs in the treatment and control arms at baseline (i.e., before consumption of the test protein/placebo) are given in Table 2. At baseline, there was no significant difference between the treatment and control arms for any of the EAA parameters. The carryover effect during the crossover trial was assessed. There was no significant carryover effect between the two periods for any of the EAA parameters (*P* > 0.05).

### 3.2. Effect of Protein Supplement/Placebo on Essential Amino Acid Levels

**Figure 2:**
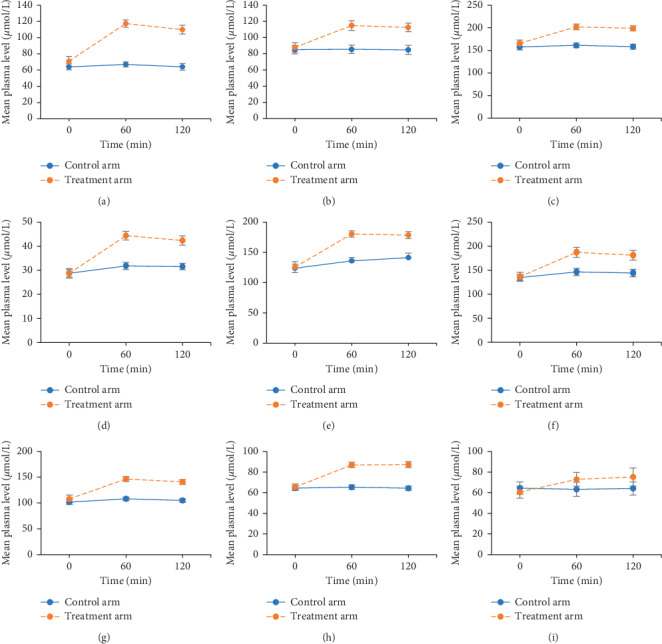
Changes in plasma levels of essential amino acids with time in the treatment and control arms. (a–c) Branched-chain amino acids. (d–i) Other essential amino acids. (a) Isoleucine. (b) Leucine. (c) Valine. (d) Methionine. (e) Lysine. (f) Threonine. (g) Tryptophan. (h) Phenylalanine. (i) Histidine.

**Figure 3:**
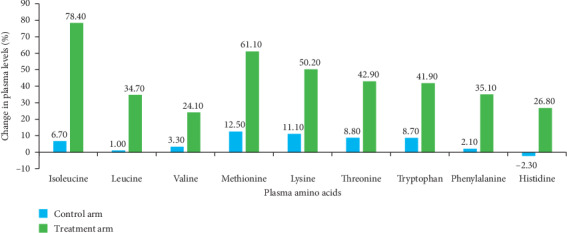
Percentage changes in plasma levels of essential amino acids from baseline to 60 min in the treatment and control arms.

A comparison of the change in plasma levels for all EAAs with time in the treatment and control arms is presented in Figure 2. The efficacy of the treatment for the various amino acids was assessed and results revealed that all the amino acids showed significant time effect and interaction (time × treatment) effect in the treatment arm. The mean levels of all the amino acids increased significantly (*P* < 0.01) from baseline to 60 min, with no significant change from 60 to 120 min. The levels peaked at 60 min (except for histidine and phenylalanine, which peaked at 120 min) but continued to remain higher even at 120 min. In the control arm, mean amino acid levels did not vary significantly across the three time points. The magnitude of increment for all amino acids from 0 to 60 min was significantly greater in the treatment arm as compared with the control arm (time × treatment arm; interaction *P* < 0.01 for all amino acids). However, the sequence effect and sequence by treatment interaction were not significant for any of the outcomes. The percentage change from baseline to 60 min was calculated for the treatment and control arms (Figure 3). The percentage change in plasma levels from baseline to 60 min was greater in the treatment arm as compared with the control arm. The BCAAs valine, leucine, and isoleucine demonstrated percentage increments of 24%, 35%, and 78%, respectively, in the treatment arm. Other EAAs, including histidine, phenylalanine, tryptophan, threonine, lysine, and methionine, showed percentage increments ranging from 27% to 61% in the treatment arm, while in the control arm, the changes in the levels of these amino acids ranged from −2.3% to 12.5%. In addition, the percentage increments from baseline to 60 min in the treatment arm for EAAs including leucine, isoleucine, threonine, histidine, tryptophan, and lysine showed a significant positive correlation with age (correlation coefficient, 0.54–0.60). The correlation was the strongest for isoleucine. Although statistically not significant, the mean percentage increment in the treatment arm was higher among females compared with males.

Reported as mean ± standard error (error bars denote standard error calculated for the 10 participants in each arm); *P* values obtained using repeated measures ANOVA to assess the effect of treatment on amino acid levels.

## 4. Discussion

The data from the current study indicated that following a dose of protein, the concentration of EAAs was significantly higher in the treatment arm in comparison to the control arm. The increase achieved in the control arm may have been due to the milk component of the feed. It was observed that consumption of the protein supplement resulted in increments of 24%, 35%, and 78% in plasma levels of the BCAAs valine, leucine, and isoleucine, respectively. For the other EAAs, including histidine, phenylalanine, tryptophan, threonine, lysine, and methionine, the percentage of increment ranged from 27% to 61%.

An optimal balance of amino acids in the diet and circulation is crucial for whole body homeostasis. Besides being building blocks of proteins, some of the amino acids also play important roles in the regulation of key metabolic pathways that are necessary for maintenance, growth, reproduction, and immunity [26]. When foods that contain protein are consumed, amino acids appear in the bloodstream at rates influenced by the composition of the food. These amino acids then gradually disappear from circulation as they are taken up by different tissues [27]. The rate of disappearance from the circulation depends on the uptake by extraintestinal organs and tissues especially skeletal muscle as it is considered to be responsible for most of the uptake of plasma amino acids [28]. The source of the essential amino acids, that is, dietary protein derived (DPEAAs) versus free-form EAAs (FEAAs), could affect the essential amino acid kinetics [29]. While those with compromising nutrient absorptive capacity (chronic disease, cancer, etc.) or nutritionally high risk (elderly) may greatly benefit from consuming FEAAs, dietary derived DPEAAs are still an effective nutrition mode among healthy adults. [30, 31]. The current study used DPEAAs and demonstrated that there was a substantial increment in circulating EAAs levels. Thus, DPEAAs supplementation in daily diet could act as a potential feasible option for healthy Indian young adults. The abundant availability of all EAAs is known to act as a stimulant for muscle protein synthesis. In the postfed state, EAAs act as precursors for muscle protein synthesis. The higher plasma concentrations of EAAs resulting from the digestion of the consumed protein could provide the required substrate for muscle protein synthesis [32]. The fact that, in our study, the circulating levels of EAAs were higher in the treatment arm than in the control arm is promising in this direction. Indians are generally considered to be of a thin frame with low muscle mass compared with Caucasians [33]. The concept of thin-fat Indian phenotype is evolving of late, as a majority of Asian Indians develop type 2 diabetes despite having a low or normal BMI [34]. With preexisting low muscle mass, it is important to further understand the role of nutrients, especially protein intake with high EAAs, in the diet among Indians with type 2 diabetes.

In the present study, isoleucine showed the highest plasma amino acid levels among all the BCAAs. The BCAAs leucine, isoleucine, and valine are the key nutrient regulators of muscle protein synthesis [7]. Leucine is considered to be a specific stimulator of protein synthesis in several tissues apart from skeletal muscle, including adipose tissue [35] and liver [36]. The stimulatory effect of leucine on protein synthesis occurs at the level of translation and involves signaling through the mammalian target of rapamycin (mTOR) [36]. Animal studies have shown that in addition to protein synthesis, isoleucine, a positional isomer of leucine, prevents a rise in the plasma glucose concentration. The effect of isoleucine was greater than that of leucine or valine in an oral glucose tolerance test in normal rats. The fact that isoleucine plays a role in glucose homeostasis adds an additional advantage over and above its role in protein synthesis. With the increasing prevalence of type 2 diabetes in India, the role of targeted supplementation, such as an isoleucine-enriched diet, could drive future studies. Isoleucine is believed to reduce plasma glucose levels through a signaling pathway involving phosphatidylinositol-3-kinase (PI3K) and protein kinase C (PKC). It is suggested that isoleucine assumes the role of a signal for glucose metabolism, thereby stimulating insulin and mTOR-independent glucose uptake by skeletal muscle cells [37]. Although we did not explore the glucose and insulin response to EAAs in the current study, this could form the basis for future studies to uncover the role of EAAs, especially isoleucine, in the prevention of type 2 diabetes.

During exercise, amino acid oxidation, protein breakdown, and suppression of protein synthesis occurs. Postexercise availability of an adequate pool of amino acids facilitates protein synthesis to maintain muscle homeostasis [38, 39]. There is some evidence that the basal plasma amino acid levels differ markedly between healthy but sedentary individuals and those who are physically active [40, 41]. Einspahr and Tharp reported that plasma levels of leucine, isoleucine, and tyrosine were 41%, 27%, and 23% higher, respectively, in athletes who ran 110 km/week on average than in untrained individuals who ran less than 5 km/week [41]. The Indian Council of Medical Research-India Diabetes (ICMR-INDIAB) study, one of the largest studies exploring the physical activity levels and prevalence of type 2 diabetes in India, revealed that the majority of Indians (>50%) in both rural and urban regions were sedentary [42]. With the present study population being sedentary, the observed increments in the circulating pool of EAAs with a dose of protein supplement are promising. One limitation of this study is that we evaluated only the acute effect of protein supplementation. The chronic effects of protein supplementation on the amino acid pool, as well as the long-term physiological effects, were not investigated. This calls for future studies to understand the role of chronic exercise with protein supplementation on muscle mass and function, including fitness, among Indians. The second limitation of the study is related to the fact that multiple data points would have allowed us to explore and understand the utilization aspect of the essential amino acids. However, the current study was limited by logistic and budget constraints. Future studies could use this study as a basis to explore prolonged sampling protocol.

## 5. Conclusion

The present study showed that consumption of a protein supplement added to milk resulted in significantly greater increments in the circulatory amino acid pool than the consumption of milk alone. This study is the first to use the targeted EAA analysis approach to investigate the effect of protein supplementation on plasma levels of EAAs among healthy Asian Indians. The increments in plasma levels of all EAAs, especially isoleucine, observed in our study are promising and provide a rationale for future studies to evaluate the role of EAAs in conjunction with exercise in both healthy and diseased individuals, especially those with type 2 diabetes.

## Figures and Tables

**Table 1:** Baseline characteristics of the study participants.

Parameters	Values
Age (years)	28.6 ± 6.4
Height (m)	1.62 ± 0.09
Weight (kg)	60.6 ± 9.9
BMI (kg/m^2^)	22.9 ± 2.63
*Body composition*	
Total fat percentage (%)	33.7 ± 7.1
Total lean mass (kg)	38.5 ± 8.0
Appendicular muscle mass (kg)	23.8 ± 6.1
BMD total (g/cm^2^)	1.12 ± 0.10
Android fat (kg)	1.54 ± 0.49
*Questionnaire data*	
Physical activity level	1.51 ± 0.13
Total energy intake per day (kcal)	1747.3 ± 362.5
Percentage of calorie intake from protein (%)	12.7 ± 1.2
Percentage of calorie intake from carbohydrate (%)	56.7 ± 5.9
Percentage of calorie intake from fat (%)	30.1 ± 5.4
*Biochemical tests*	
Fasting blood sugar (mg/dL)	85.7 ± 7.7
HbA1c (%)	5.3 ± 0.2
Cholesterol (mg/dL)	167 ± 39
Triglycerides (mg/dL)	66 (46, 119)

BMI: body mass index; BMD: bone mineral density; HbA1c: glycated hemoglobin. Data are presented as mean ± SD or median (interquartile range).

**Table 2:** Plasma levels of essential amino acids at baseline in the treatment and control arms.

	Control arm (*μ*mol/L)			Treatment arm (*μ*mol/L)		
	Minimum	Maximum	Mean ± SD	Minimum	Maximum	Mean ± SD
Isoleucine	36.2	109.6	63.9 ± 16.5	38.9	160.1	70.9 ± 26.9
Leucine	49.4	121.4	85.0 ± 23.3	46.8	144.5	87.8 ± 26.2
Valine	101.2	209.6	157.7 ± 29.5	105.6	222.9	165.8 ± 31.9
Methionine	16.9	40.5	28.8 ± 6.8	19.0	55.6	28.7 ± 8.7
Lysine	78.9	208.8	123.9 ± 29.7	75.0	274.4	125.6 ± 40.7
Threonine	98.8	188.2	134.7 ± 27.6	81.9	202.1	136.3 ± 41.2
Tryptophan	62.1	134.6	101.8 ± 20.7	65.2	216.2	107.9 ± 32.8
Phenylalanine	41.6	84.1	64.5 ± 10.3	51.0	112.6	65.5 ± 13.7
Histidine	25.1	117.7	64.4 ± 26.7	21.6	124.4	60.5 ± 26.6

SD: standard deviation.

## Data Availability

The data used to support the findings of this study are available from the corresponding author upon request.

## Supplementary Materials

Supplementary Materials Nutritional composition and list of ingredients of the test protein supplement and placebo.
