# Supplementary material for: Acute Effect of a Protein Supplement on Targeted Plasma Amino Acid Profile among Healthy Asian Indians: A Randomized Controlled Trial
Source: J Nutr Metab. 2020 May 27;2020:8946820. doi: 10.1155/2020/8946820 (PMC7285395; doi:10.1155/2020/8946820)
Supplement: Supplementary Materials — Nutritional composition and list of ingredients of the test protein supplement and placebo. [file 8946820.f1.pdf]

| Nutritional Composition | | | | | |
|---|---|---|---|---|---|
| **Nutrients** | **Unit** | **Test protein supplement** | | **Placebo** | |
| | | **Per 100 g** | **Per serve (35 g)** | **Per 100 g** | **Per serve (35 g)** |
| Energy | kcal | 366 | 128 | 388.0 | 135.8 |
| Carbohydrate | g | 54.0 | 18.9 | 95.8 | 33.5 |
| Protein | g | 34.0 | 11.9 | 1.0 | 0.4 |
| Fat | g | 1.5 | 0.53 | 0.1 | 0.0 |
| Calcium | mg | 1300.0 | 475 | | |
| Iron | mg | 20.0 | 7.6 | | |
| Phosphorous | mg | 900.0 | 348 | | |
| Magnesium | mg | 50.0 | 22.8 | | |
| Zinc | mg | 3.0 | 1.1 | | |
| Iodine | mg | 73.0 | 29.8 | | |
| Copper | mg | 400.0 | 149.4 | | |
| Sodium | mg | 400.0 | 157 | | |
| Potassium | mg | 870.0 | 286 | | |
| Chloride | mg | 360.0 | 148 | | |
| Choline | mg | 107.1 | 37.5 | | |
| Vitamin A | mcg | 1142.9 | 400 | | |
| Vitamin B1 | mg | 2.3 | 0.8 | | |
| Vitamin B2 | mg | 3.4 | 1.2 | | |
| Vitamin B6 | mg | 1.1 | 0.4 | | |
| Vitamin B12 | mcg | 0.9 | 0.3 | | |
| Niacin | mg | 35.7 | 12.5 | | |
| Pantothenic acid | mg | 2.3 | 0.8 | | |
| Biotin | mcg | 23.7 | 8.3 | | |
| Vitamin C | mg | 60.0 | 21.0 | | |
| Folic acid | mcg | 119.4 | 41.8 | | |
| Vitamin D | mcg | 6.6 | 2.3 | | |
| Vitamin E | mg | 12.3 | 4.3 | | |

| Amino acid profile of test protein supplement | | |
|---|---|---|
| **Amino acids (g)** | **Per 100 g** | **Per serve (35 g)** |
| Isoleucine | 1.706 | 0.5971 |
| Leucine | 2.955 | 1.03425 |
| Valine | 1.861 | 0.65135 |
| Methionine | 0.624 | 0.2184 |
| Lysine | 2.344 | 0.8204 |
| Threonine | 1.394 | 0.4879 |
| Tryptophan | 0.515 | 0.18025 |
| Phenylalanine | 1.768 | 0.6188 |
| Histidine | 0.888 | 0.3108 |

| List of ingredients | |
|---|---|
| **Test protein supplement** | **Placebo** |
| Skimmed milk powder, sucrose, soy protein isolate, wheat flour, stabilizer (guar gum), maltodextrin, corn flour, vitamins (choline bitartrate, ascorbic acid, nicotinamide, dl-alpha tocopheryl acetate, retinyl acetate, riboflavin, ergocalciferol, thiamine hydrochloride, calcium pantothenate, pyridoxine hydrochloride, folic acid, d-biotin), malt extract, minerals (ferric pyrophosphate, zinc sulphate, potassium iodide) | Maltodextrin, sucrose, flavor, cereal mix |
